# Electrospun multifunctional nanofibrous mats loaded with bioactive anemoside B4 for accelerated wound healing in diabetic mice

**DOI:** 10.1080/10717544.2021.2021319

**Published:** 2022-01-03

**Authors:** Hao Zhang, Mengyao Zhang, Xumei Wang, Mi Zhang, Xuelian Wang, Yiyang Li, Zhuoer Cui, Xiuping Chen, Yantao Han, Wenwen Zhao

**Affiliations:** a Qingdao University Medical College, Qingdao, China; b State Key Laboratory of Quality Research in Chinese Medicine, Institute of Chinese Medical Sciences, University of Macau, Macao, China

**Keywords:** Traditional Chinese medicine, anemoside B4, inflammatory, diabetic wound healing

## Abstract

With the worldwide prevalence of diabetes and considering the complicated microenvironment of diabetic wounds, the design and development of innovative multifunctional wound dressing materials are much wanted for the treatment of hard-to-heal wounds in diabetic patients. In the present study, anti-inflammatory ingredients loaded with nanofibrous wound dressing materials were manufactured by a promising blend-electrospinning strategy, and their capability for treating the diabetic wound was also systematically explored. A polymer blend consisting of Chitosan (CS) and polyvinyl alcohol (PVA) was electrospun into CS-PVA nanofibrous mats as control groups. In the meanwhile, a bioactive ingredient of Chinese medicine *Pulsatilla*, anemoside B4(ANE), with different contents were loaded into the electrospinning solution to construct CS-PVA-ANE nanofibrous mats. The developed CS-PVA-ANE wound dressing materials exhibited multifunctional properties including prominent water absorption, biomimetic elastic mechanical properties, and sustained ANE releasing behavior, as well as outstanding hemostatic properties. The *in vitro* studies showed that the CS-PVA-ANE nanofiber mats could significantly suppress lipopolysaccharide (LPS)-stimulated differentiation of pro-inflammatory (M1) macrophage subsets, and notably reduce the reactive oxygen species (ROS) generation, as well as obviously decrease inflammatory cytokine release. The *in vivo* animal studies showed that the CS-PVA-ANE nanofiber mats promoted the healing of diabetic wounds by significantly enhancing wound closure rates, accelerating excellent angiogenesis, promoting re-epithelization and collagen matrix deposition throughout all stages of wound healing. The present study demonstrated that CS-PVA-ANE nanofiber mats could effectively shorten the wound-healing time by inhibiting inflammatory activity, which makes them promising candidates for the treatment of hard-to-heal wounds caused by diabetes.

## Introduction

Nowadays, the incidence of diabetes is rapidly rising due to the changes in diet and lifestyle, accelerated economic development, population aging, and urbanization (Smokovski et al., 2015). According to the statistics, the prevalence of diabetes can increase to 439 million persons (7.7% of the global population), by 2030 (Shaw et al., 2010). Compared with diabetes itself, some complications associated with diabetes, including heart attack (Harding et al., 2019), stroke (Zhou et al., 2019), blindness (Han et al., 2019), amputation (Pérez-Panero et al., 2019), and kidney failure (Bakris et al., 2019), pose a greater threat to human life. Noticeably, the hard-to-heal wounds are one of the most severe diabetic complications, ultimately resulting in limb loss and disability. More than 750,000 cases of diabetic foot ulcer (DFU) occur in the USA every year, and ∼10% of these require amputation surgeries of lower limbs (den Dekker et al., 2019). Wound dressings have been extensively employed to treat diabetic wounds in clinics. Importantly, the recent advances have improved the healing efficacy of conventionally-utilized wound dressing materials by incorporating some bioactive ingredients into the dressing materials, containing growth factors (Bienert et al., 2017; Laiva et al., 2018; Vijayan et al., 2019) and exosomes (Tao et al., 2017; Shiekh et al., 2020; Zhao et al., 2020), which have been demonstrated to accelerate endothelial proliferation and angiogenesis. Unfortunately, some demerits, such as the instability of additives and the exorbitant price, severely hold back their clinical application (Matoori et al., 2021).

From a pathological point of view, the common wound healing is a well-orchestrated process, referring to several defined phases including hemostasis, inflammation, proliferation, and remodeling (Boniakowski et al., 2017). Unfortunately, the healing process of diabetic wounds is different from the common wounds, which are usually disrupted and blocked by prolonged low-grade inflammation, consequentially slowing down the wound healing (Eming et al., 2014). Therefore, suppressing the inflammatory response is recognized as a critical factor for promoting wound healing in the early inflammatory stage (Li et al., 2020). In addition, the wound healing process requires the necessary involvement of macrophages (Kim & Nair, 2019). During the normal wound healing process, the macrophages undergo a transition from pro-inflammatory (M1) to anti-inflammatory (M2), which facilitates angiogenesis, granulation tissue formation, and wound closure (Kotwal & Chien, 2017). However, for diabetic wounds, the dysregulation of the M1/M2 ratio in the wound sites usually causes the wound healing process into a self-perpetuating inflammatory phase, further leading to intractable and impaired wound healing issues (Barman & Koh, 2020). Worse still, the long-term inflammation presence can assuredly suppress the migration and proliferation of endothelial cells (Iba & Levy, 2018; Dalal et al., 2020) and further limit the generation of blood vessels. Therefore, The addition of anti-inflammatory agents into wound dressings is a promising strategy for the treatment of chronic diabetic wounds.

In our study, electrospun nanofiber mats were chosen as the wound dressing materials, which are expected to better mimic the morphology and structure of the collagen fibrils existing in the natural skin extracellular matrix (ECM) (Keshvardoostchokami et al., 2020). Moreover, the electrospinning mats have been widely demonstrated to promote various cell activities, such as adhesion (Kopec et al., 2020; Zha et al., 2020), proliferation (Fu et al., 2017; Dadashpour et al., 2018) of human dermal fibroblasts, and accelerate the regeneration and healing of wound sites in various animal models. Importantly, the electrospinning mats were also employed as a drug delivery carrier to load the ANE, which is one of the main active ingredients from traditional Chinese herbal *Pulsatilla*. ANE has been previously reported to ameliorate LPS-induced ear swelling (Kang et al., 2019), attenuate cisplatin-induced acute kidney injury (Gong et al., 2019), and protect against *Klebsiella pneumoniae*-induced pneumonia in mice (Li et al., 2020). The addition of ANE into the electrospinning nanofiber mats was expected to construct one multifunctional wound dressing, which could effectively release the anti-inflammatory ANE ingredient in the wound sites to enhance diabetic wound healing and skin regeneration.

## Materials and methods

### Fabrication of CS-PVA and CS-PVA-ANE nanofiber mats

Twenty milligrams of Chitosan (CS-MW-160 kDa and 75–85% deacetylated) and 20 mg polyvinyl alcohol (PVA-MW-146 kDa and 99% hydrolyzed) were dissolved in 3 mL acetic acid and 1 mL deionized water, respectively. The two solutions were fully mixed for the subsequent electrospinning process. For the preparation of ANE-contained CS and PVA solution, 1 mL of ANE solution with the four different concentrations, i.e. 0.1, 0.2, 0.3, and 0.4 (w/v), was added into the above-mentioned CS and PVA solutions, respectively.

The as-prepared five different solutions were electrospun into nanofiber mats. During electrospinning, a voltage of 15 kV (Chungpa EMT Co. Ltd., Seoul, Korea; model CPS-60K02VIT) was applied to the syringe needle, which was connected with the spinning solution contained syringe. A syringe pump was utilized to fix the solution flow rate at 0.5 mL/h. A grounded Al foil was employed to collect the prepared nanofibers, the distance between the needle and collector was 150 mm. The needle size (length = 20 mm, diameter = 1 mm) was controlled on the spinneret for electrospinning. The electrospinning was performed at conditions of 25 °C and 60% relative humidity. Samples I–V were utilized to name all the five different nanofiber mats. The sample I stands for the nanofiber mats prepared from pure CS and PVA, while Samples II–V stand for the nanofiber mats with ANE incorporated at 0.1–0.4 (w/v).

### Scanning electron microscopy (SEM)

The morphology of the fibers was determined by SEM (TESCAN VEGA3, Czech Republic) at an accelerating voltage of 15 kV. All samples were sprayed with gold for 90 s before the observation.

### Contact angle measurement and swelling ratio

Samples were cut into a size of 2 × 2 cm^2^. The water contact angle was measured with a contact angle analyzer (KRUSS DSA25, Germany). Each sample was laid flat on a glass slide fixed onto the testing platform. A drop of distilled water about 2 mm in diameter was dripped onto the sample surface, and the contact angles were measured at 1, 2, 3, 4, and 5 s after contact of the drop with the sample. Each measurement was repeated six times, and the average value was calculated.

The swelling ratio of the samples was measured by immersing weighed samples into distilled water for 24 h at room temperature. The original weight of the samples and the weight after water absorption for 24 h were defined as *w_o_* and *w_s_*, respectively. The swelling ratio was calculated according to the following equation:

$$Sw\mathrm{ellingRatio}=\frac{ws-wo}{wo}\times 100\%$$





### *In vitro* release curve of ANE from nanofiber mats

An *in vitro* elution method was used to analyze the release behavior of ANE from the nanofiber mats containing different concentrations of ANE. 10 × 10 mm sample was placed in a 1 mL buffer solution and incubated in a 37 °C constant temperature shaker. At present time intervals, a part of the incubation liquid was withdrawn for UV spectrophotometry measurements of the ANE released into the buffer solution.

### Mechanical properties

The mechanical properties of nanofiber mats were measured with a universal tensile machine (Instron-3300, USA). Samples were prepared with a width of 2 mm, a gauge length of 20 mm, and a thickness of 0.1–0.2 mm. Tensile strength was tested using 500 N load cells at a speed of 10 mm/min.

### Evaluation of hemostatic capability

Hemostasis by the CS-PVA-ANE mats was evaluated *in vivo* using a mouse model of hepatic hemorrhage (Wang et al., 2020). The mice (C57BL/6J mice, female, six-week-old, weight of 22–26 g) were randomly and equally divided into three groups: control group, CS-PVA group, and CS-PVA-ANE group. Each mouse was anesthetized by intraperitoneal injection of 4% w/v chloral hydrate (0.01 mL/g mouse body weight). The mouse liver was exposed through an abdominal incision, removed, and transferred to a dry pre-weighed filter paper. The liver was dissected, followed by a puncture with an 18 G needle (1.2 mm) to initiate bleeding. Nanofiber mats were immediately applied to the surface of the trauma site. The control group was not treated after tying the liver. The hemorrhaging site of the liver was photographed at 0, 5, 15, 30, and 60 s.

### Cell cytotoxicity

The cell culture medium was prepared by immersing 3 mg different samples in a 1 mL commercialized cell culture medium for 24 h at 37 °C, respectively (Mirzaeei et al., 2021). The L929 cells were seeded at a density of 1 × 10^4^ cells/well in a 96-well plate and cultured for 24 h. Experiments were initiated by replacing the commercial culture medium in each well with 100 µL of sample-prepared culture medium and incubated at 37 °C in a CO_2_ incubator. Cell viability was quantitatively measured by the CCK-8 assay. The gross morphology of the cells was determined by calcein-AM staining (10 µM, 15 min) and propidium iodide (5 µM, 15 min) and observed with a confocal microscope (Nikon, Japan).

### ELISA assays

RAW264.7 cells were provided by the Institute of Chinese Academy of Sciences (Beijing, China). The cells were cultured in Dulbecco’s modified Eagle’s medium (DMEM) containing 10% fetal bovine serum and 1% penicillin/streptomycin at 37 °C in 5% CO_2_. RAW264.7 cell samples were pretreated with CS-PVA or CS-PVA-ANE for 1 h and then stimulated with 1 μg/mL LPS for 12 h. The cell supernatants were collected and tested for the presence of inflammatory cytokines using ELISA kits.

The skin wound tissues were also isolated from diabetic mice on day 3, milled in cold PBS, and then centrifuged at 12,000 rpm for 10 min at 4 °C to collect supernatants. The concentrations of the cytokines IL-6 and TNF-α in the supernatants were determined using ELISA kits.

### Flow cytometry analysis

RAW264.7 cells were pretreated with CS-PVA or CS-PVA-ANE for 1 h and stimulated with 1 μg/mL LPS for 6 h. Then treated cells were stained with FITC-conjugated antibody against CD80 (for detecting M1) and Cy5-conjugated antibody against CD206 (for detecting M2). All antibodies were prepared in a fluorescence-associated cell sorting buffer. The immunophenotypic analyses were conducted by incubating cell suspensions (2 × 10^6^ cells/mL) incubated with 10% goat serum in the dark (4 °C, 15 min), followed by incubation with antibodies for 30 min. Flow cytometry was performed on a BD LSR II (BD Biosciences, San Jose, CA, USA) and analyzed using FlowJo software (Tree Star, San Carlos, CA, USA). Each experiment was performed in triplicate.

### Establishment of a diabetic full-thickness wound in an animal model

All animal protocols were approved by the ethics committee of Qingdao University. The BALB/c mice (8-week-old, 21–26 g) used in this study were kept in a room at 24 °C with a 12-h light and dark cycle. A type 1 diabetes model was induced by fasting the mice overnight, followed by an intraperitoneal injection with 1% streptozotocin (STZ, 65 mg/kg) dissolved in 0.1 M sodium citrate buffer (pH 4.5). Five days later, whole-blood glucose obtained from the tail vein was monitored using a glucose meter (Johnson, China). After five weeks of recording, mice with constant blood glucose levels above 16.7 mM, along with the observed weight loss and polyuria, were considered diabetic. The diabetic mice were anesthetized with chloral hydrate, and the hair on their backs was completely removed with depilation cream. A standardized full-thickness wound 10 mm in diameter was created on the exposed skin. The wounded mice were randomized into four groups: a control group (no treatment), a CS-PVA group (treated with CS-PVA only), a CS-PVA-ANE group (treated with the CS-PVA-ANE mats), and a BFX™ group (treated with Recombinant Bovine Basic Fibroblast Growth Factor Gel, one widely applied external material for wound healing, as a positive control).

### Wound-healing examination

Digital photos were taken to monitor diabetic wounds at days 0, 3, 6, 9, and 12, and the diabetic wound size was calculated using the following formula:

$$\text{Percent wound size reduction }(\%)\;=\;\frac{{\mathrm{Day}}_{0}-{\mathrm{Day}}_{x}}{{\mathrm{Day}}_{0}}\times 100\%$$

where the initial area of the wound was marked as Day_0_. The area of the wound on day *x* (*x* ≥ 1) was marked as Day*_x_*. The mice were sacrificed on day 7 or day 14 after surgery.

### Histology and immunofluorescence

The skin re-epithelialization process in diabetic mice was characterized by histological staining of wound tissue sections. Skin tissues removed from the wound bed of the mice sacrificed at day 7 and day 14 were fixed in 4% paraformaldehyde for 24 h. The fixed tissues were embedded in paraffin, sectioned at a thickness of 5 μm, and stained with H&E and Masson trichrome stains.

For immunofluorescence analysis, paraffin sections of the wound tissue samples from the control, CS-PVA, CS-PVA-ANE, and BFX™ groups were first dehydrated, boiled in sodium citrate buffer for about 20 min, and then incubated with antibodies to Ki67 (4A Biotechnology Co., Ltd., China) and CD31 (4 A Biotechnology Co., Ltd., China) at 4 °C for 2 h. The sections were washed three times with PBS and then sealed with an anti-fluorescence quenching tablet containing 4′,6-diamidino-2-phenylindole (DAPI, Solarbio, China). The slides were examined with a fluorescence microscope (Nikon A1 MP, Japan). Quantitative analysis was performed using Image J software.

### Western blotting

Total proteins were extracted from wound tissues, and the protein contents were quantified with a BCA Protein Assay Kit. About 30 μg of protein was subjected to SDS-PAGE and transferred onto a PVDF membrane. After blocking with 10% non-fat milk in TBST (20 mM Tris-HCl, 500 mM NaCl, and 0.1% Tween 20) at room temperature for 2 h, the PVDF membranes were incubated with primary antibodies including CD31, Ki67, phospho-IKKβ/IKKβ, phospho-NF-κB/NF-κB, and GAPDH (Cell Signaling Technology, USA) (1:1000) overnight at 4 °C, followed by secondary antibodies (Cell Signaling Technology, USA) (1:10,000). The protein–antibody complexes were detected with an ECL Advance Western blotting detection kit. The intensity of the bands was quantified using Quantity One software (Bio-Rad).

### Statistical analysis

All data are presented as mean ± *SD*. Data were analyzed by analysis of variance (ANOVA), followed by a *post-hoc* Tukey comparison. For two-group comparisons, two-tailed unpaired *t*-tests were used to determine statistical significance. *p* < .05 was considered statistically significant.

## Results and discussion

### Physicochemical characterization of CS-PVA and CS-PVA-ANE nanofiber mats

**Figure 1.**
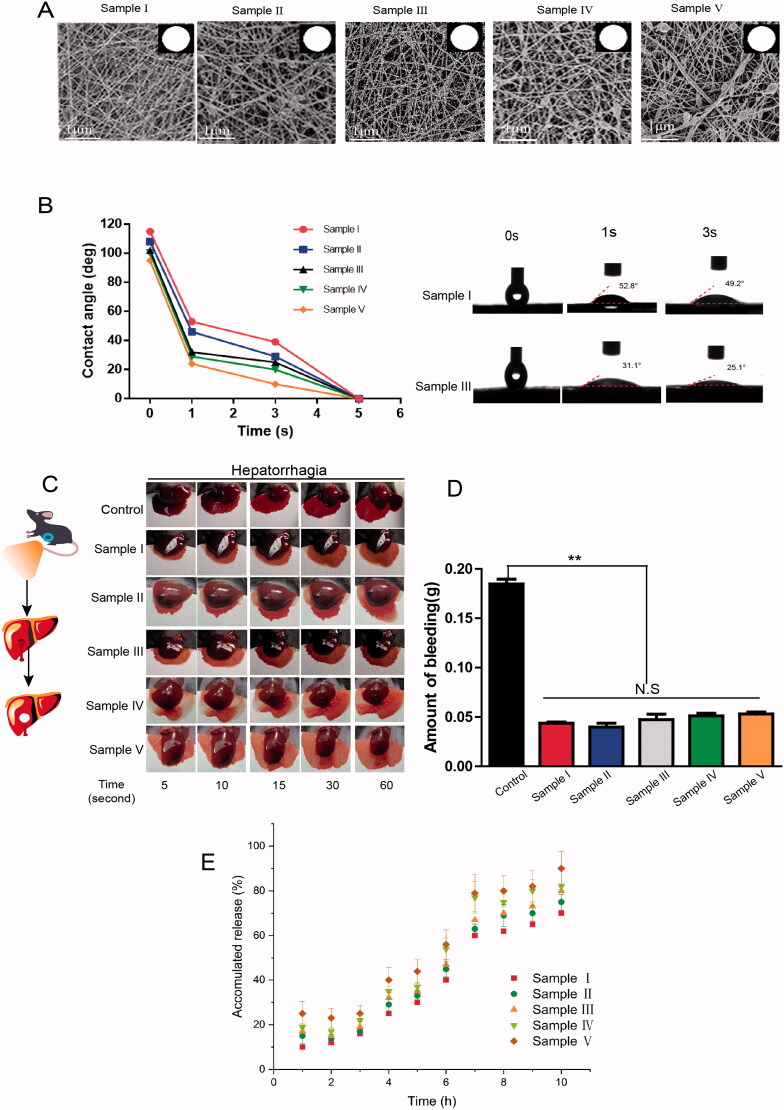
Physicochemical structural characterization of the nanofibers. (A) The SEM images of samples loaded in different concentrations: Sample I was pure PVA and CS; Samples II to V contained increasing concentrations of ANE; (B) Examination of the water absorption rate, as reflected by the change in instantaneous contact angle of the nanofibers; (C) The hemostasis of the samples was evaluated using a conventional mouse model of hepatic hemorrhage; (D) Total blood loss from the damaged livers after 60 s treated with samples and control (without treatment); (E) Drug release curves of samples loaded with ANE at different mass concentrations *vs.* control (*n* = 5; mean ± *SD*). *p*-Values: **p < .01; N.S. (no significant difference) *p* > .05. Sample I: CS-PVA; Samples II: CS-PVA-0.1 ANE; Sample III: CS-PVA-0.2 ANE; Sample IV: CS-PVA-0.3 ANE; Sample V: CS-PVA-0.4ANE.

The optimum ratio of CS-PVA to ANE was determined. As shown in Figure 1(A), Sample III was relatively homogeneous compared with the other samples and had a diameter ranging from 150 to 250 nm.

Generally, the surface hydrophilicity, hemostasis, slow-release properties, and tensile properties are vital properties for the development of wound dressing materials. The hydrophilic properties of the samples were characterized by measuring the water contact angles and swelling ratios. The water contact angle study showed that the instant contact angle (ICA) of the nanofiber membranes sharply decreased from 110 ± 10° to 0° in 5 s, indicating efficient water absorption (Figure 1(B)). The water absorption capacity of the samples was enhanced after loading with ANE (Supplementary Figure 1(A)), indicating that the nanofiber mats could prevent the accumulation of exudates in the wound bed (Li et al., 2011). These findings were further confirmed in the mouse model with liver hemorrhage. Blood loss after 60 s was significantly lower in the Sample I group (32.7 mg), compared to the Sample II group (33.4 mg), the Sample III group (39.8 mg), the Sample IV group (40.4 mg) and the Sample V group (41.6 mg), which were all obviously lower than the control group without ANE (183.1 mg) (Figure 1(C,D)). Here, the hemostatic ability was attributed to the presence of chitosan in the dressing, as this material causes red blood cells to agglutinate, and it activates platelet aggregation (Chen et al., 2018).

Maintaining a sustained and stable release of drug is also a very important clinical index for a dressing, as it can allow reductions in the dose frequency and improve patient compliance (Cam et al., 2020). The release curve of ANE from the CS-PVA-ANE mats was established spectrophotometrically by UV absorbance at the previously established maximum absorption wavelength of 285 nm (Supplementary Figure 1(B)). As shown in Figure 1(E), a 10 h release curve into PBS was obtained for ANE from the nanofibers, with no explosive release in any of the samples, confirming a persistent and steady release of ANE.

The mechanical properties of the samples were characterized by tensile measurements, which showed a gradual decrease in the tensile strength and elongation of the nanofiber mats with increasing ANE concentrations (Supplementary Figure 1(C)). However, with the selected dose of ANE, the dressing remained relatively elastic and optimal for wound healing.

In terms of desirable physical properties, the fiber morphology of Sample III was deemed more homogeneous than the others. We, therefore, selected Sample III for subsequent experiments, with Sample I serving as the control. Sample III and Sample I are henceforth referred to as CS-PVA-ANE and CS-PVA throughout the rest of the study.

Overall, the multifunctional properties of CS-PVA nanofiber dressings, including water absorption, mechanical properties, stable drug release, and favorable electrical conductivity, as well as efficient hemostatic properties, showed the promise of this dressing for applications in wound healing.

### Compatibility, antioxidative stress activity, and anti-inflammatory efficacy of CS-PVA-ANE

**Figure 2.**
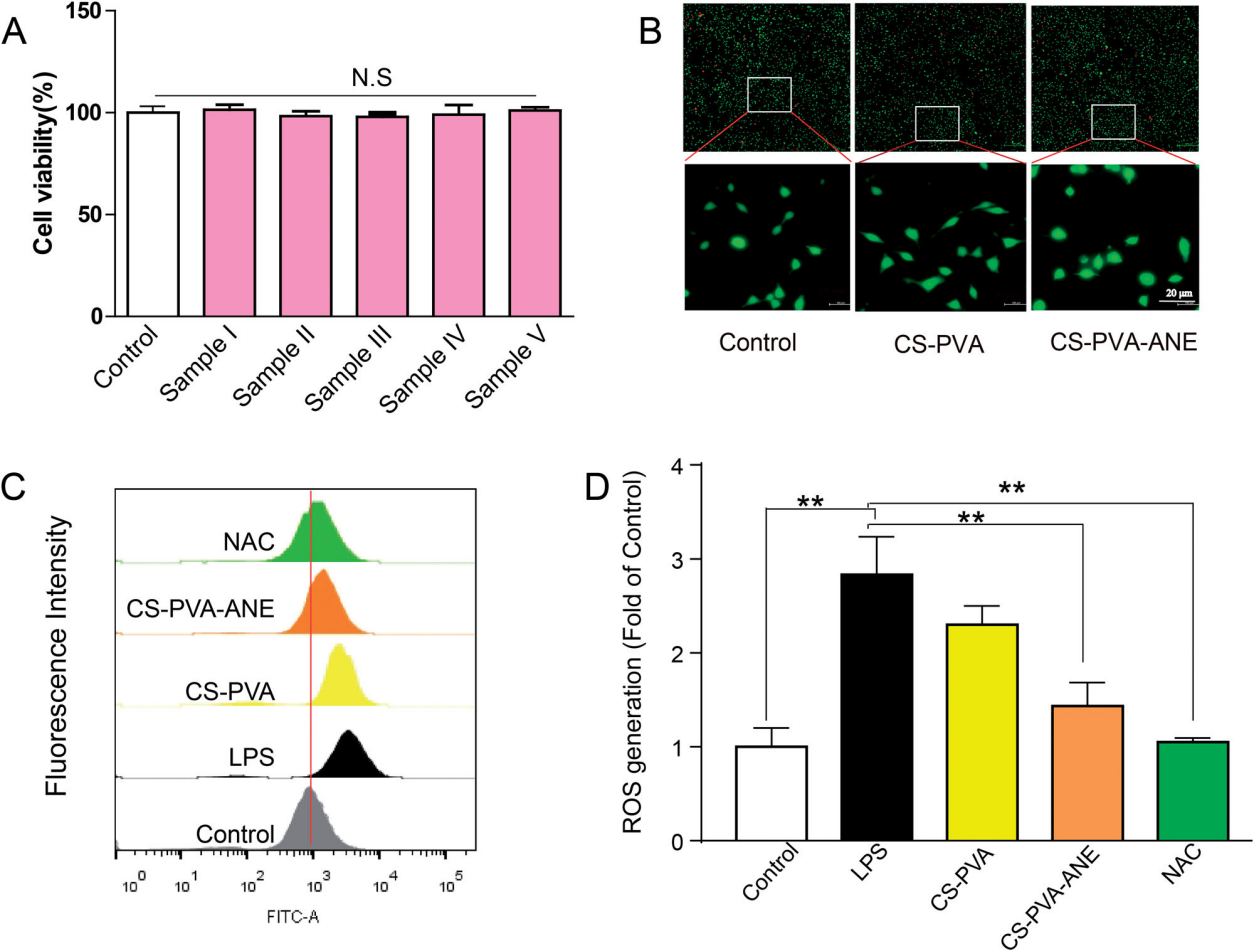
Compatibility and antioxidative stress efficiencies of CS-PVA-ANE. (A) Cell viability of L929 cells treated with different nanofiber membranes for 24 h; (B) Live/Dead staining of L929 cells after treatment with nanofiber membranes for 24 h; (C,D) ROS generation was measured by flow cytometry and quantified (*n* = 3; mean ± *SD*). *p*-Values: **p < .01; N.S. (no significant difference) *p* > .05. LPS: lipopolysaccharide; NAC: N-acetyl-L-cysteine.

The cell compatibility of CS-PVA-ANE was evaluated by both MTT assay and Live/Dead staining. As shown in Figure 2(A), no cell cytotoxicity was evident between all the experimental groups and the control group, and cell viability was higher than 98%. Live/Dead staining showed that the number of dead cells (labeled with red fluorescence) in all three groups was almost the same. Besides, the magnified image presented normal morphology (labeled in green fluorescence) in all groups demonstrated good compatibility of nanofibers (Figure 2(B)).

Considering that persistent excessive reactive oxygen species (ROS) accumulation in the wound sites is the main reason for chronic wound healing (Dunnill et al., 2017), the antioxidant activity of ANE was determined *in vitro*. Large amounts of ROS were produced in response to LPS stimulation in L929 cells, as determined with the dichlorodihydro-fluorescein diacetate (DCFH-DA) probe, while obvious fluorescence quenching was observed in L929 cells treated with the positive drug N-acetyl-L-cysteine (NAC) or with CS-PVA-ANE, indicating the outstanding radical scavenging properties of ANE (Figures 2(C,D)).

**Figure 3.**
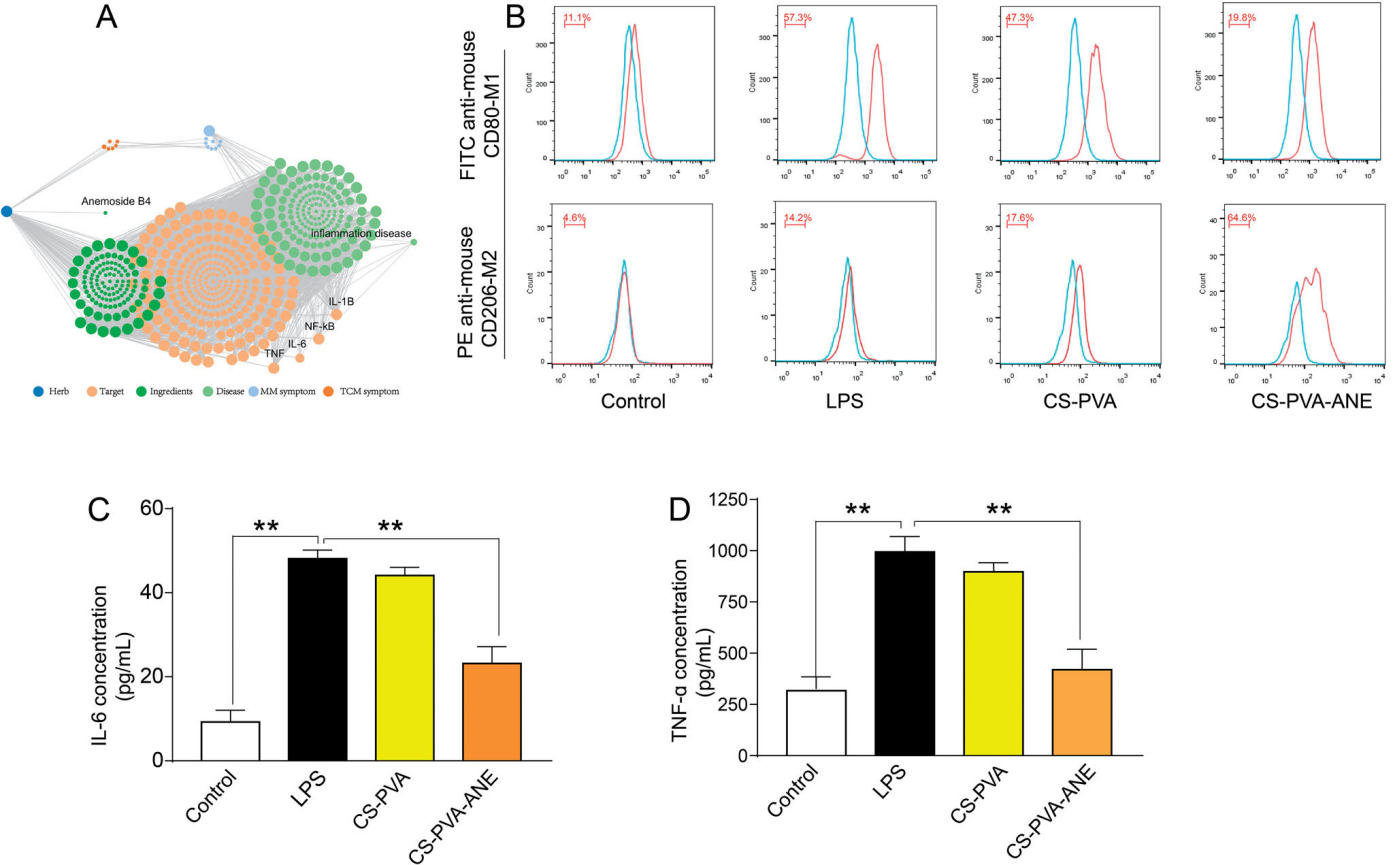
Anti-inflammatory efficacy of CS-PVA-ANE. (A) Network pharmacological chart; (B) Flow cytometry pattern of macrophages stained with CD206 and CD80 antibody after treatment with LPS and different nanofiber membranes; (C,D) Concentrations of pro-inflammatory chemokines IL-6 and TNF-α in the supernatant of macrophages determined by ELISA. LPS: lipopolysaccharide (*n* = 3; mean ± *SD*). *p*-Values: **p < .01.

Diabetic wounds show a persistent inflammatory phase that does not subside due to the continuous presence of pro-inflammatory chemokines and inflammatory cells (Joshi et al., 2020). Notably, macrophages play an important role during the tissue remodeling process and can polarize into M1 macrophages and M2 macrophages (Wicks et al., 2014). Wound dressings with the capability to promote the M1-to-M2 phenotype transition are beneficial for chronic wound healing. Our network pharmacologic analysis predicted that ANE has anti-inflammatory potential (Figure 3(A)).

We evaluated macrophage polarization by flow cytometry to reveal the *in vitro* anti-inflammatory effect and mechanisms of CS-PVA-ANE. LPS stimulation induced RAW 264.7 cells to assume the M1 phenotype, and these cells were used as the positive control (the percentage of the M1 phenotype was 57.3%). Flow cytometry analysis showed that 19.8% of the M0 macrophages differentiated into the M1 phenotype, while 64.6% differentiated into the M2 phenotype after pretreatment with CS-PVA-ANE supernatant Figure 3(B). These results demonstrated the effectiveness of CS-PVA-ANE nanofibers in promoting the transformation of macrophages from the M1 phenotype into the M2 phenotype that possesses potent anti-inflammatory properties.

Macrophages mainly display the pro-inflammatory M1 phenotype and produce a series of inflammatory cytokines that induce tissue destruction and organ dysfunction (Hu et al., 2020). The RAW 264.7 cells were pretreated with supernatant loaded with degradative material and then stimulated with LPS. After 24 h, the IL-6 and TNF-α levels in the supernatant were measured. As shown in Figures 3(C,D), LPS stimulated the release of pro-inflammatory chemokines (49.7 pg mg^−1^ for IL-6 and 1039.7 pg mg^−1^ for TNF-α), but this release was significantly reduced by CS-PVA-ANE application (22.6 pg mg^−1^ for IL-6 and 389.3 pg mg^−1^ for TNF-α), compared to the control group. This finding confirmed that CS-PVA-ANE can inhibit inflammatory responses and accelerate the healing process by suppressing the secretion of pro-inflammatory cytokines. The previous studies also demonstrated that the macrophage polarization from M1 to M2 would effectively enhance wound repair (Wu et al., 2021).

### CS-PVA-ANE accelerates cutaneous wound healing in diabetic mice

**Figure 4.**
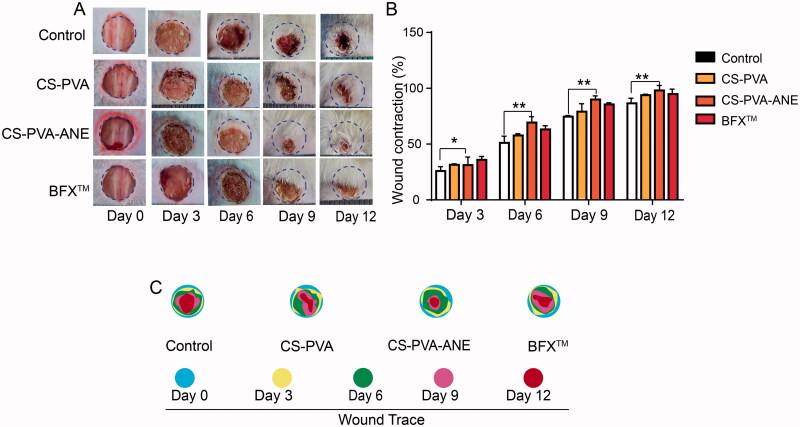
Healing process of diabetic wounds promoted by treatment with a CS-PVA-ANE nanofiber membrane. (A) Representative images of wound-healing process of mice with different treatments; (B) Traces of wound-bed closure during 12 days for each treatment; (*n* = 6; mean ± *SD*) (C) Wound closure rates at different time points of four groups. *p*-Values: *p < .05, **p < .01.

CS-PVA-ANE clearly possessed multifunctional properties, especially referring to anti-inflammatory and antioxidant bioactivity, which showed its promise in diabetic wound healing. We examined the wound-healing capability of CS-PVA-ANE in a mouse model with a full-thickness diabetic wound. The CS-PVA and CS-PVA-ANE dressings, as well as BFX™ (a commercial positive control), were applied to process full-thickness diabetic wounds, and the diabetic wound sizes were photographed at days 0, 3, 6, 9, and 12 post-operatively. As shown in Figure 4(A), the wound surface in all four groups decreased markedly as time increased. The CS-PVA-ANE group exhibited the best healing outcomes, showing complete closure and an inconspicuous scar on day 12. By contrast, the wounds in the CS-PVA, BFX™, and control groups were still exposed and covered by eschar.

Quantitative analysis of the wound-healing rate was consistent with the gross observations. On day 6, the wound-healing rate for the CS-PVA-ANE nanofiber mats group was 41.2%, which was significantly higher than that of the control group (25.2%) and that of the CS-PVA nanofiber mats group (32.6%). After 12 days of treatment, the wound healed completely in the CS-PVA-ANE nanofiber group, for a significantly higher healing rate (97.8%) than those observed in the control group (71.4%) and the CS-PVA nanofiber mats group (84.5%) (Figure 4(B)).

### Histomorphological analysis and in vivo angiogenesis

**Figure 5.**
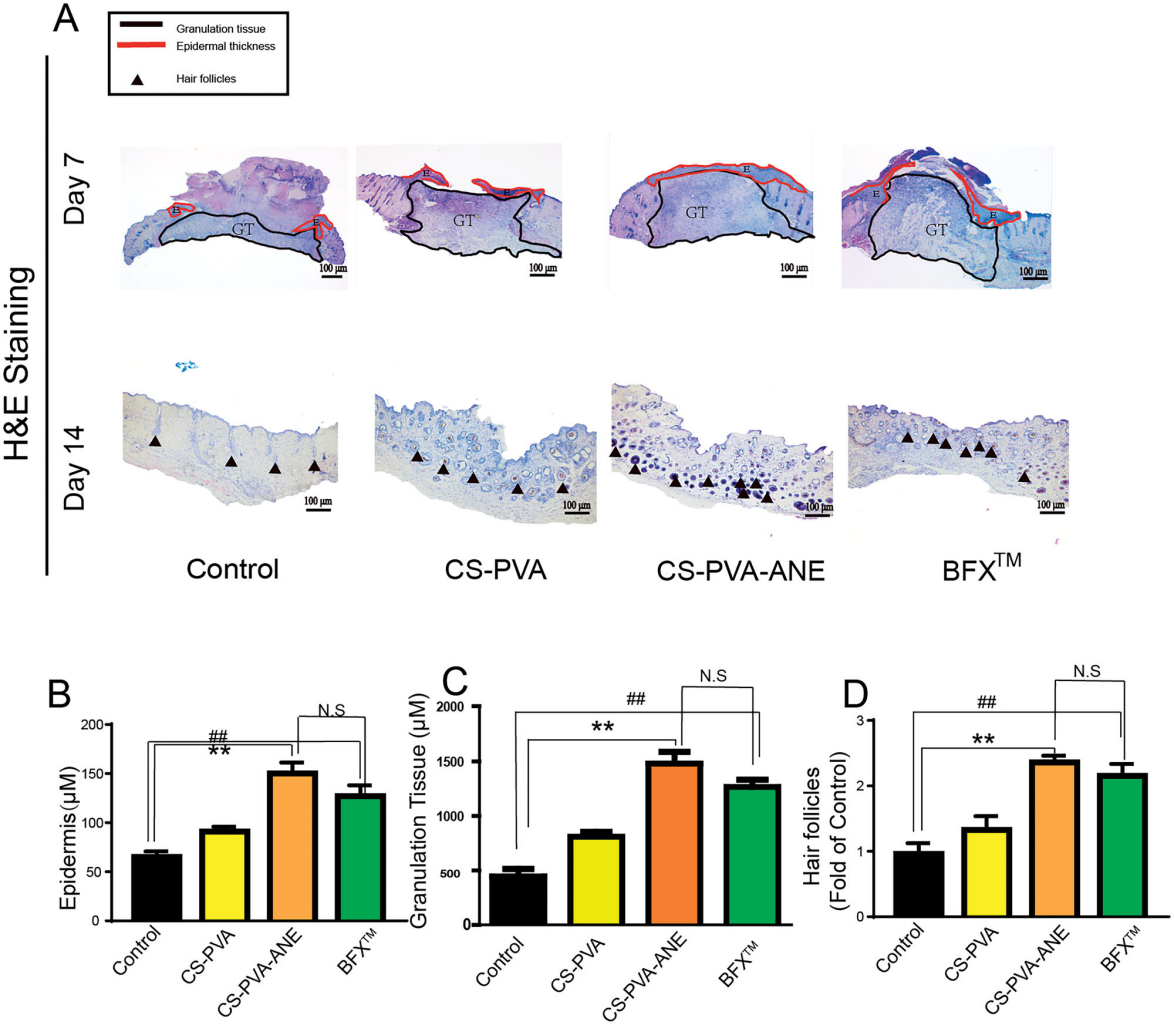
Histological evaluation of diabetic wound healing. (A) H&E staining of wound site on days 7 and 14; GT: granulation tissue; E:epidermial thickness; (B) The thickness of epidermis; (C,D) The thickness of granulation tissue and hair follicles were quantified. Control group, diabetic wound without treatment; CS-PVA group, diabetic wound was treated with nanofibers; CS-PVA-ANE group, diabetic wound treated with ANE (*n* = 6; mean ± *SD*). *p*-Values: **p < .01; *^##^p* < .01; N.S. (no significant difference) *p* > .05.

Histopathology is an intuitive means to monitor wound-healing progress and assess morphological changes (Masson-Meyers et al., 2020). We evaluated the changes in wound-healing pathology by H&E staining and Masson trichrome staining. In the early stage, the epidermis was thickened due to the proliferation and differentiation of endothelial cells (Vagnozzi et al., 2015). As shown in Figure 5(A), the results illustrated that the control group formed a few neo epidermises on day 7, and meanwhile, more granulation tissue and epidermis-like cell arrangement formed in the wound sites of both CS-PVA and CS-PVA-ANE and BFXTM groups. Clearly, the degree of tissue regeneration in the CS-PVA-ANE group was better than the other three groups. Chen’s previous study demonstrated that the dressing made from CS could act as a barrier to avoid further damage and infection of the wound site (Chen et al., 2018). Therefore, the healing rates of the wounds in the CS-PVA group were better than those in the control group. In addition, newly formed granulation tissue and epidermis were thicker and better structured than other groups, suggesting more rapid healing of the wounds when treated with CS-PVA-ANE (Figures 5(B,C)). In the late stage, the number of hair follicles reflected the degree of recovery of the wound. By day 14, the CS-PVA-ANE group had far more numerous hair follicles than the other groups (Figure 5(D)).

**Figure 6.**
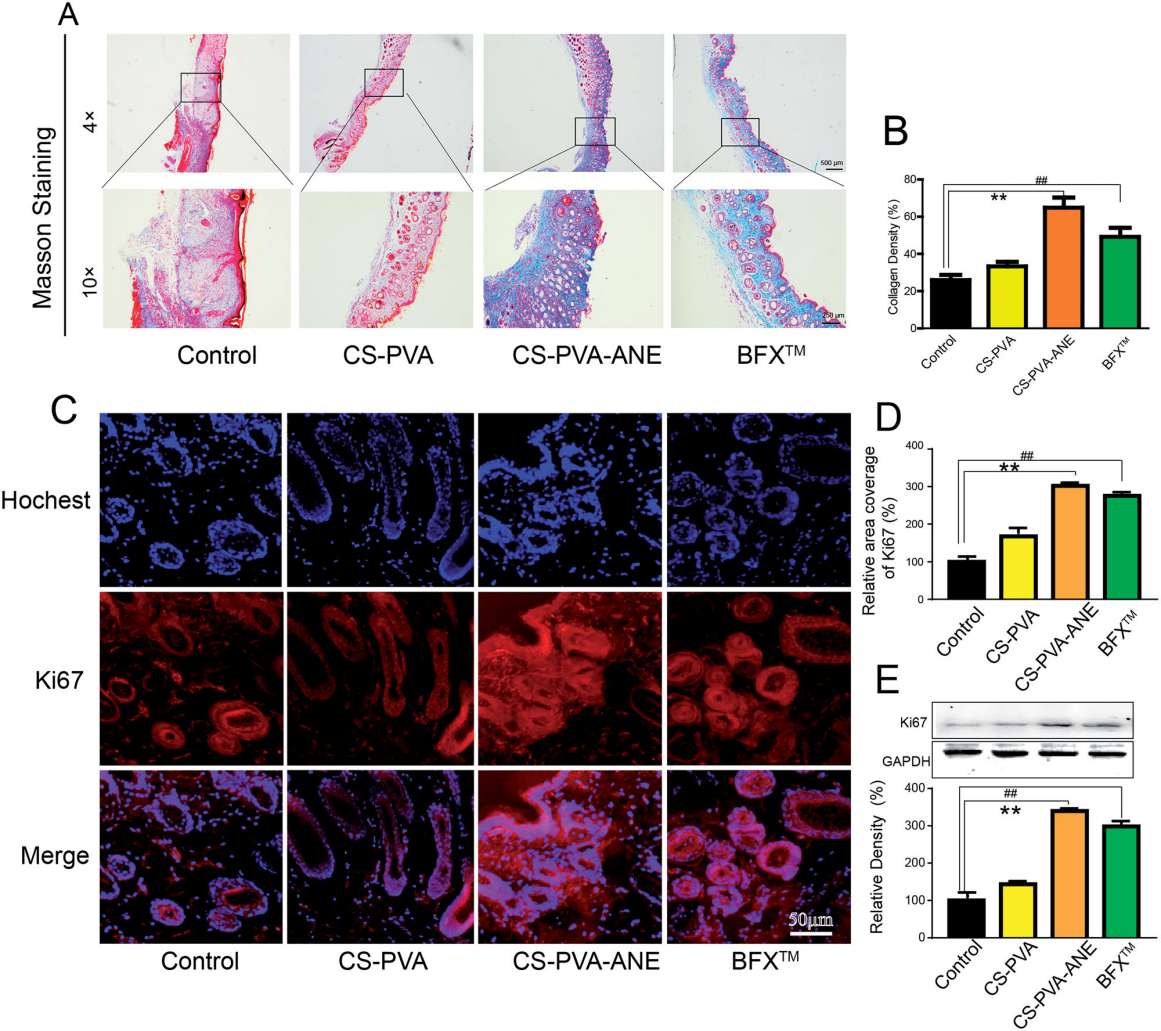
CS-PVA-ANE accelerated wound healing in the late wound healing stage. (A) On the 14th day, the deposition of collagen fibers in the wound was observed at low power and high power; (B) Quantification of collagen density after Masson's trichrome staining; (C,D) Immunofluorescence staining of Ki67 in the wound bed on day 14; (E) Protein expression levels of Ki67 determined by western blotting (*n* = 6; mean ± *SD*). *p*-Values: **p < .01; *^##^p* < .01.

During the process of wound healing, collagen deposition plays an important role in determining skin scar formation and improving tissue strength in the late stage (Koudouna et al., 2020). Masson trichrome staining was performed to determine the deposition of nascent collagen in the regenerated skin tissue on day 14. As shown in Figure 6(A,B), the relative intensity of collagen fibers stained with blue color was highest in the CS-PVA-ANE group.

**Figure 7.**
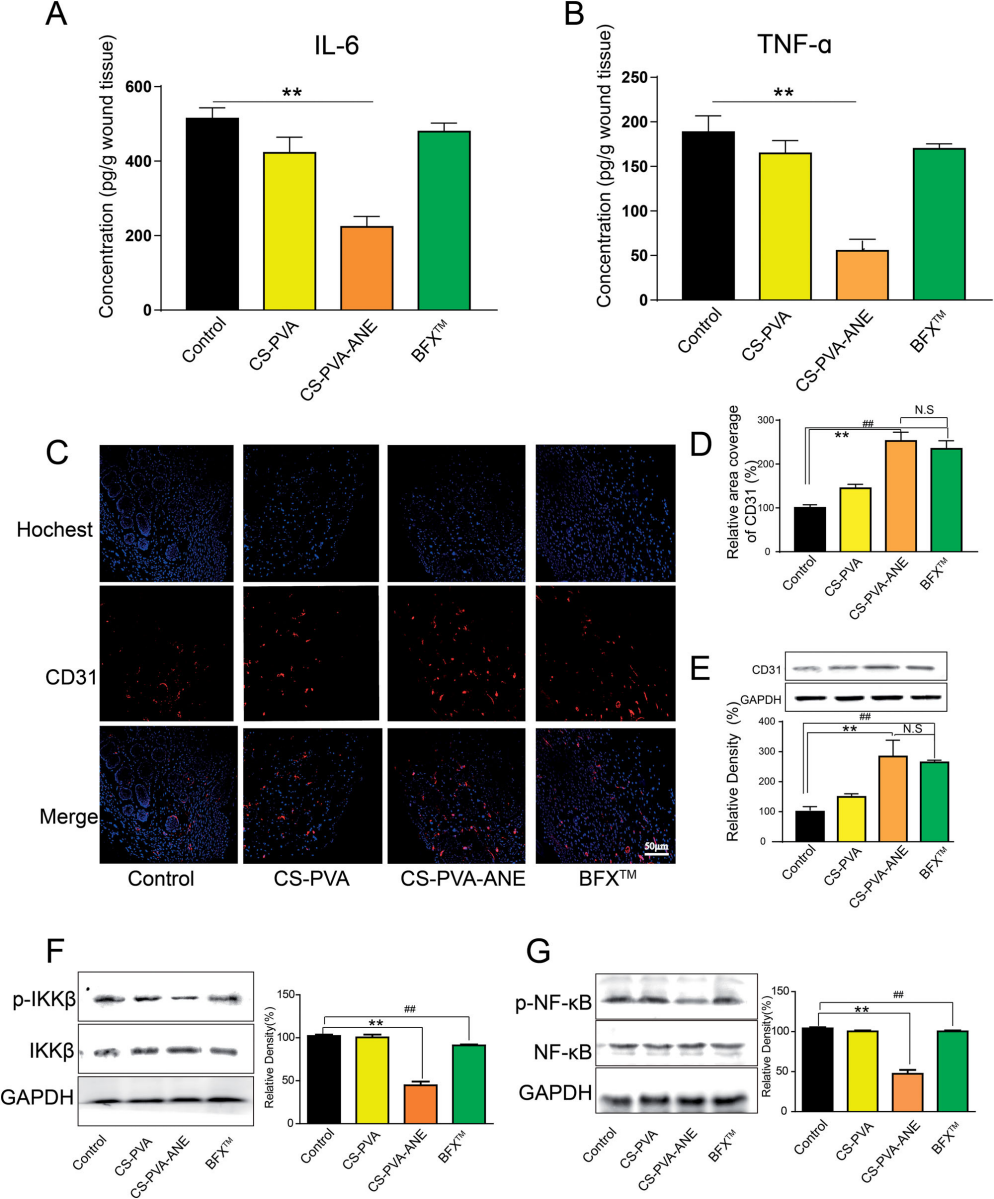
ANE modulated the inflammation microenvironment to promote chronic diabetic wound healing. Wound tissues were harvested at day 3 after the different treatments, and the concentrations of chemokines IL-6 (A) and TNF-α (B) in wound tissues were determined by ELISA. Immunofluorescence images of CD31 (red) and nuclei (blue) in skin wound tissues on day 14 (C) and (D) Quantitative analysis of the relative coverage area; (E–G) Proteins in wound tissues were isolated and detected by western blotting (*n* = 6; mean ± *SD*). *p*-Values: **p < .01; ^##^*p* < .01; N.S. (no significant difference) *p* > .05.

Ki-67 is a marker of cell proliferation (Chang et al., 2010). In our study, the cell proliferation in the wound tissues was determined by the expression of Ki-67. The immunofluorescence staining showed that the intense red fluorescence (label Ki67) was detected in the CS-PVA-ANE group, while the intensity and relative areas were much lower in the control group (Figure 6(C,D)). The results from Western blotting further confirmed the high expression of Ki67 in the wound areas treated with CS-PVA-ANE (Figure 6(E)), hinting that the addition of ANE accelerated organizational reconstruction.

### CS-PVA-ANE modulated the inflammatory microenvironment to promote angiogenesis

An inflammatory microenvironment is assuredly pivotal during the process of wound healing and tissue regeneration (Chazaud, 2014). The inflammation phase in chronic wounds is severely prolonged, which even stops the transition into the proliferation phase (Makrantonaki et al., 2017). In our *in vitro* study, ANE obviously suppressed the release of TNF-α and IL-6 inflammation-related cytokines as well as effectively reduced the generation of ROS. Consistent with *in vitro* study, in *in vitro* experiment, Tissues on the wound area on day 3 in mice were isolated and the release of chemokines in the wound tissues was detected. As shown in Figure 7(A), a large release of IL-6 from the focal area occurred in the control group (450 pg g^−1^) but was significantly reduced with CS-PVA-ANE treatment (210 pg g^−1^). Similar results were found for TNF-α (190 pg g^−1^ for the control group, 170 pg g^−1^ for the CS-PVA group, 55 pg g^−1^ for the CS-PVA-ANE group, and 175 pg g^−1^ for the BFX™ group) (Figure 7(B)). These results suggested that the CS-PVA-ANE nanofiber mats could reduce the inflammatory response in chronic diabetic wounds in the early stage of wound healing by reducing pro-inflammatory factors.

It has been reported that the transformation of the wound from inflammation to proliferation can be accelerated to maintain a stable remodeling state of diabetic chronic wounds and enhance the healing effect. Angiogenesis is the result of the proliferation of endothelial cells, which provide nutrients and oxygen for wound healing to accelerate wound repair. To further explore the potential regulation of anti-inflammation on angiogenesis, we validated the formation of blood vessels by immunofluorescent staining of the endothelial cell marker CD31 (Figure 7(C,D)). Besides, CD31 protein tested by Western blotting was highly expressed in the wound area in the CS-PVA-ANE group (Figure7(E)). These results are consistent with our previous anti-inflammatory results and suggest that CS-PVA-ANE may promote angiogenesis by shortening the inflammatory period. It has been reported that M2 macrophages can secrete vascular endothelial growth factor (VEGF) to promote the proliferation of endothelial cells (Liu et al., 2020). We speculate that the CS-PVA-ANE may promote the transformation of macrophages from pro-inflammatory M1 to reparative M2 phenotype and further promote the proliferation of endothelial cells.

The IKKβ/NF-κB pathway, as a proinflammatory pathway, plays a pathogenic role in various inflammatory diseases. These cytokines including IL-6, TNF-α, and IL-1β are downstream targets of the IKKβ/NF-κB pathway (Singh & Singh, 2020). Besides, CD31 was downregulated with NF-κB activation (Baker et al., 2011). In our study, protein expressions of IKKβ and NF-κB were detected. As shown in Figure 7(F,G), phosphorylations of IKKβ and NF-κB were observed in the control group, CS-PVA group, and BFX™ group while CS-PVA-ANE significantly suppressed IKKβ and NF-κB activity. Our study hinted that IKKβ/NF-κB may act as the potential upstream players for regulating inflammatory cytokines and angiogenesis factors.

## Conclusion

A multifunctional CS-PVA-ANE nanofiber membrane was successfully developed and showed excellent air permeability, good water absorption, adequate mechanical properties, stable drug release properties, as well as efficient hemostatic properties. The incorporation of ANE could impart the nanofiber mats with broad biological activities, including compatibility, antioxidant, and anti-inflammatory properties, which could accelerate cutaneous wound healing in a mouse model of diabetic wounds. Compared to the CS-PVA nanofiber membrane alone, the CS-PVA-ANE nanofiber membrane shortened the inflammatory period and prolonged the cell proliferation and organizational reconstruction period by suppressing inflammatory responses. Overall, the CS-PVA-ANE membrane shows great potentials as a multifunctional wound dressing material for full-thickness diabetic wound repair.

## Supplementary Material

Supplemental Material
