# Supplementary material for: Electrospun multifunctional nanofibrous mats loaded with bioactive anemoside B4 for accelerated wound healing in diabetic mice
Source: Drug Deliv. 2022 Jan 3;29(1):174–85. doi: 10.1080/10717544.2021.2021319 (PMC8725929; doi:10.1080/10717544.2021.2021319)
Supplement: Supplemental Material [file IDRD_A_2021319_SM5312.doc]

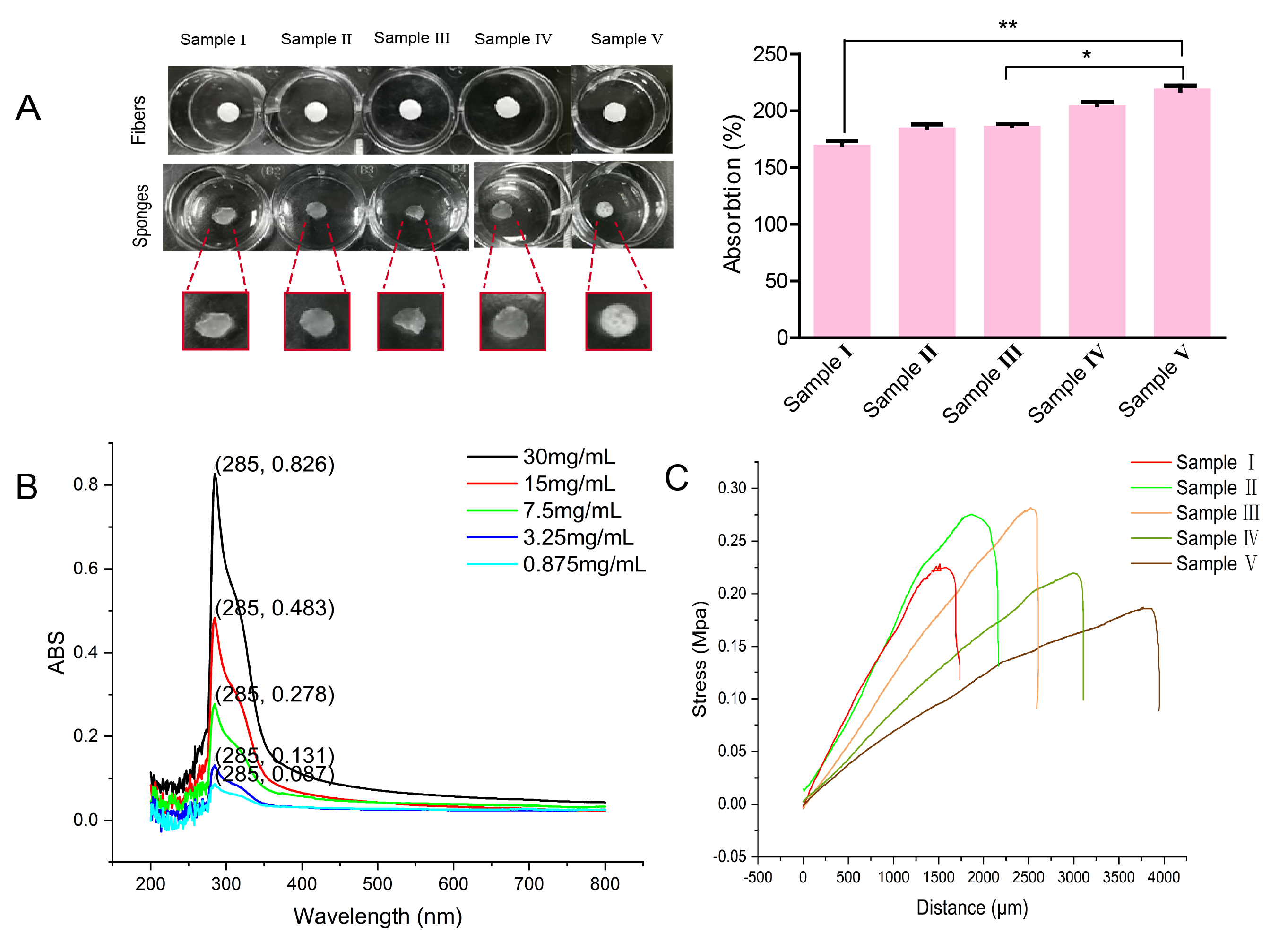


# Supplementary

Figure 1. (A) Water absorption of nanofiber mats loaded with ANE of different mass concentrations; (B) Ultraviolet absorption spectra of different concentrations of ANE; (C) Typical tensile stress-strain curves of samples. (n=3; mean ± SD). *P* values: **p* < .05, ***p* < .01, versus Sample Ⅰ.
